# Developmental Regulation and Induction of Cytochrome P450 2W1, an Enzyme Expressed in Colon Tumors

**DOI:** 10.1371/journal.pone.0122820

**Published:** 2015-04-06

**Authors:** Eva Choong, Jia Guo, Anna Persson, Susanne Virding, Inger Johansson, Souren Mkrtchian, Magnus Ingelman-Sundberg

**Affiliations:** Department of Physiology and Pharmacology, Section of Pharmacogenetics, Karolinska Institutet, Stockholm, Sweden; Nihon University School of Medicine, JAPAN

## Abstract

Cytochrome P450 2W1 (CYP2W1) is expressed predominantly in colorectal and also in hepatic tumors, whereas the levels are insignificant in the corresponding normal human adult tissues. CYP2W1 has been proposed as an attractive target for colorectal cancer (CRC) therapy by exploiting its ability to activate duocarmycin prodrugs to cytotoxic metabolites. However, its endogenous function, regulation and developmental pattern of expression remain unexplored. Here we report the CYP2W1 developmental expression in the murine and human gastrointestinal tissues. The gene expression in the colon and small intestine commence at early stages of embryonic life and is completely silenced shortly after the birth. Immunohistochemical analysis of human fetal colon revealed that CYP2W1 expression is restricted to the crypt cells. The silencing of CYP2W1 after birth correlates with the increased methylation of CpG-rich regions in both murine and human CYP2W1 genes. Analysis of CYP2W1 expression in the colon adenocarcinoma cell line HCC2998 revealed that the gene expression can be induced by e.g. the antitumor agent imatinib, linoleic acid and its derivatives. The imatinib mediated induction of CYP2W1 suggests an adjuvant therapy to treatment with duocarmycins that thus would involve induction of tumor CYP2W1 levels followed by the CYP2W1 activated duocarmycin prodrugs. Taken together these data strongly support further exploration of CYP2W1 as a specific drug target in CRC.

## Introduction

Colorectal cancer (CRC) is an important contributor to cancer mortality and morbidity with limited treatment options for patients with advanced disease. CRC causes half a million deaths annually and the general five years survival rate is around 60% [1]. Currently, surgical resection remains the most curative approach for CRC, but approximately 40% of treated patients will subsequently develop local recurrence or distant metastases [2,3]. Therefore, refinement of the strategies for the treatment of CRC metastases is of crucial importance.

The orphan cytochrome P450 2W1 (CYP2W1) has been found to be expressed preferentially in colorectal tumors while its expression is barely detectable in non-transformed tissues [4]. High CYP2W1 levels were observed in approximately 30% of human CRC specimens [5,6]. Recently significant expression has also been seen in liver cancers with even higher expression in liver metastases than in the parent tumor from the same patient [7]. In both colon and liver cancers the prognosis of survival is worse in patients carrying tumors with higher levels of CYP2W1 expression [5,6,8]. Low expression levels of CYP2W1 are observed also in the other tumors, e.g. adrenal gland, gastric and lung tumors [4,9,10].

Tumor-specific expression of CYP2W1 has been suggested as an attractive target for CRC therapy exploiting either its ability to activate certain prodrugs to cytotoxic metabolites or using CYP2W1 as a new tumor-associated antigen for immunotherapy-based treatment. Thus far, the prodrug monotherapy (PMT) option has been successfully confirmed both *in vitro* and in a murine xenograft model [11].

In this context, the exploration of the mechanisms that control CYP2W1 expression is of paramount importance as it might lead to the development of a novel combined therapy of CRC that would include a tumor-specific induction of CYP2W1 followed by the treatment with CYP2W1-specific prodrugs, increasing thus the number of patients who would benefit from such approach.

In addition to its expression in colon cancer cells, the CYP2W1 mRNA has been reported in rat fetal colon and in murine embryonic pooled tissues, whereas no significant expression has been reported in adult tissues. However, the developmental expression of human CYP2W1 remains largely unexplored. In CRC tumors, higher expression was associated with increased demethylation of CpG island in the exon 1/intron 1 junction [9]. This implies interesting parallels between the developmental and cancer regulatory mechanisms of CYP2W1 expression that were also found to overlap in many oncofetal genes [12,13].

There have been limited attempts to modulate the intracellular levels of CYP2W1. Previous unpublished study suggested CYP2W1 induction by imatinib in leukaemia cells (GEO dataset: GDS3044) and CYP2W1 was induced in breast cancer cells following the treatment with 5F-203 or GW-610 [14]. However, the extensive analyses of CYP2W1 regulation in colon cancer cells are missing.

The aim of current study was to examine CYP2W1 regulation by (i) studying the murine and human developmental patterns of its constitutive expression and putative epigenetic regulation and (ii) screening of the potential CYP2W1 inducers that could modulate the levels of its endogenous expression.

## Materials and Methods

### Human and animal studies

Human adult and fetal tissues were obtained from the NICHD Brain and Tissue Bank for Developmental Disorders at the University of Maryland (Baltimore, MD, USA) after approval of our study by the committee of the University. Adult colon (n = 5) and small intestine (SI, n = 5) control samples were from donors between 19 and 61 years of age with heart/pulmonary failure or accidental death diagnosis. The three colon and five SI human fetal tissues were from different female donors, gestational weeks 18 and 19.

Cyp2w1 murine expression from fetal life to adulthood was analyzed by collecting C57BL/6 WT mouse colon and SI tissue samples from embryonic days 13 (E13), E16, E18, followed by postnatal day 0, day 2, 3, 7 and 28. Adult mice were investigated at 12 weeks of age, 7 and 13 months of age (n = 6 per time point). Mice were anesthetized by Ketamine/Xylazine intraperitoneally followed with CO_2_. The tissues were collected immediately after cervical dislocation and frozen in dry ice. Animal experimentation was approved by the local animal ethical committee in Stockholm, Sweden (permits: N147/11 and N505/11).

### Western blot

Human and mice tissues were homogenized using the Bullet Blender (Next Advance, Averill Park, NY, USA) in a buffer containing 10 mM Tris–HCl pH 7.5, 0.5 mM EDTA, 0.25 M sucrose with addition of protease inhibitors cocktail (Roche Diagnostics, Mannheim, Germany). HCC2998 cells were lysed in RIPA buffer containing protease inhibitors cocktail for 30 min at 4°C. Denatured protein (40 μg) samples were run on 10% SDS-PAGE, transferred to nitrocellulose membranes and immunoblotted with two different rabbit anti-human CYP2W1 antibodies (H175, Santa Cruz, CA, USA, dilution 1:200 and the C-terminal in-house anti-CYP2W1-852 [4], dilution 1:1000), rabbit anti-mouse CYP2W1 antibodies (in-house anti-CYP2W1-3675, dilution 1:500), or with rabbit anti-ERp29 (in-house antibody reacting with both species [15], dilution 1:1000),javascript:void(0); followed by goat anti-rabbit conjugated horseradish peroxidase secondary antibodies (Dako, Glostrup, Denmark, dilution 1:2000). Filters were developed using SuperSignal West Femto Chemiluminescent Substrate (Pierce, Rockford, IL, USA) and signals detected by LAS-1000 system (Fujifilm, Japan).

### CYP2W1 mRNA expression

Total RNA and DNA were extracted using AllPrep DNA/RNA Mini Kit (Qiagen, Hilden, Germany) from 10–50 mg of human and mouse colon, SI or confluent HCC2998 cells. Reverse transcription was performed using the SuperScript III first-strand synthesis (Invitrogen, Rockville, USA). Real-time polymerase chain reaction was carried out using ready-to-use CYP2W1 (Hs00908623_m1, Mm01207203_m1 for human and mice, respectively) and CYP1A1 (Hs00153120_m1) TaqMan Gene Expression Assays (Applied Biosystems, Stockholm, Sweden), according to the manufacturer's instructions. An internal control sample on the plate and housekeeping genes, EIF2B2 (Hs00204540_m1), HMBS (Hs00609293_g1) and PPIA (Hs99999904_m1) for human and Tjp1 (Mm00493699_m1), 18S (Mm03928990_g1) and Gadph (4352339E) for mice were used for normalization according to the 2^-ΔΔCt^ method [16].

### Immunohistochemistry (IHC)

Fresh human fetal colon tissue was sectioned, mounted on slides, and kept at -20°C until used. Sections were fixed in ice-cold 3.7% formaldehyde in phosphate buffered saline (PBS pH 7.4, Gibco, 14190–094) and incubated in blocking solution (3% Bovine Serum Albumin (Sigma-Aldrich, Stockholm, Sweden), 0.25% Triton X-100 (Sigma-Aldrich), 0.01% NaN3 (Merck, Sollentuna, Sweden) in PBS) followed by overnight incubation with primary antibody at 4°C. Primary antibodies used: rabbit anti-CYP2W1-H175 (Santa Cruz Biotechnology, Santa Cruz, CA, USA, 1:200 dilution, and home-made anti-CYP2W1-852 [4], 1:1000 dilution), mouse anti-GRP78 (StressGen, Victoria British Columbia, Canada, 1:500 dilution). Sections were further incubated with the following secondary antibodies: Alexa Fluor anti-rabbit 488, anti-mouse 555 (Jackson ImmunoResearch, 1:500 dilution), mounted, dried and finally covered with ProLong Gold Antifade with DAPI (Invitrogen). Images were obtained using a Zeiss 710LSM laser scanning microscope (Carl Zeiss HB, Stockholm, Sweden).

### Analyses of 5-methylcytosine

Bisulfite modified genomic DNA samples from mice between the ages of E13 to 13 months were obtained using EZ DNA Methylation-Gold Kits (Zymo Research Corp, Irvine, CA, USA). The *in silico* analysis showed enrichment of murine cyp2w1 gene by CpG sites in several areas (S1 Fig). Selected areas (n = 6) within the cyp2w1 gene and upstream region were amplified with two subsequent PCR reactions using either the Taq DNA polymerase and associated reagents (Thermo Scientific, St. Leon-Rot, Germany) or the KAPA HiFi HotStartUracil ReadyMix (Kapa Biosystems, Inc., Wilmington, USA). Same protocol was used for DNA samples from human fetal and adult colon tissue and also for the HCC 2998 cells treated with CYP2W1 inducing agents (see Cell cultures section, below). All primers were designed using the online bisulfite primer design software (http://bisearch.enzim.hu/) and optimized to amplify a single PCR product. S1 Table describes PCR conditions and primer sequences used. PCR products were then run on a 1.5% TAE gel, the bands were excised and DNA samples were purified using a QIAquick Gel Extraction kit (Qiagen). Direct Sanger sequencing (Eurofins, Ebersberg, Germany) was performed and percentage of methylated cytosines was evaluated by the peak ratio of cytosine or guanine peak height (forward or reverse primer, respectively), to the peak height sum of cytosine and thymine or guanine and adenine (forward or reverse primer, respectively) using Chromas 2.4.1 (Technelysium, South Brisbane, Australia).

### Cell cultures

The human colon adenocarcinoma HCC2998 cells obtained from National Cancer Institute (Frederick, MD, USA) were cultured in RPMI 1640 medium supplemented with 10% fetal bovine serum, 100 U/ml penicillin–100 μg/ml streptomycin, including 2 mM L-glutamine. Passage numbers between 11 and 16 were used throughout the study.

HEK 293T cells were cultured in Dulbecco’s modified Eagle’s DMEM medium with 10% fetal bovine serum, and 100 U/ml penicillin–100 μg/ml streptomycin. The pCMV6-Entry plasmid containing mouse cyp2w1 cDNA (Origene, Rockville, MD, USA), the previously described cDNA construct of human CYP2W1 [17] or an empty plasmid were transfected using 2 μg Lipofectamine 2000 for 48 h. HepG2 cells at passage 13 were used as a second positive control for human CYP2W1 [4] and were maintained in MEM medium supplemented with 10% fetal bovine serum, 100 U/ml penicillin–100 μg/ml streptomycin, 1 mM sodium pyruvate, and non-essential amino acids. All cell culture media and reagents were obtained from Life Technologies (Stockholm, Sweden).

### CYP2W1 activity

The CYP2W1 enzymatic activity was assessed by measuring the metabolism of a CYP2W1-specific non-toxic duocarmycin derivative substrate (ICT2726) as previously described [11]. Five days after reaching the confluent state HCC2998 cells were incubated with ICT2726 (4 μM) for 24 h, after which cells were washed twice with PBS and the reaction was stopped by adding 100μl of ice-cold acetonitrile. After centrifugation at 4°C, 13000 g for 20 min, 30 μl of supernatant was injected into a Luna C18 (2.0x150 mm, 5 μm col HPLC column; Phenomenex, Værløse, Denmark), as previously described [17]. The cytotoxic effect of two duocarmycin prodrugs, ICT2705 and ICT2706 [11] was tested on the confluent HCC2998 cells that were incubated for 72 h with the vehicle (0.1% DMSO) or ICT2706 and ICT2705. Cell viability was assessed by the EZ4U assay (Biomedica, Vienna, Austria) according to manufacturer´s instructions.

### Induction of the endogenously expressed CYP2W1

Drugs were selected based on literature mining of CYP2W1 induction [14], NCBI GEO database, and activity reported in some artificial recombinant models [18–20]. We have also included modulators of the known transcriptional factors, such as AhR, CAR, PXR, PPARγ, RXR, RAR (S2 Table).

Linoleic acid (LA), conjugated (9Z,11E)-linoleic acid (9Z11E-LA), conjugated (10E,12Z)-linoleic acid (10E12Z-LA), citco, rifampicine, 9-cis-Retinoic acid (RA), all-trans-retinoic acid, ciglitazone, 5-Aza-2'-deoxycytidine (DAC), 2,3,7,8-tetrachlorodibenzo-p-dioxin (TCDD) were purchased from Sigma-Aldrich (Stockholm, Sweden). Imatinib was obtained from Toronto Research Chemicals (North York, Canada) and GW610 from Cayman Chemical Comp (Ann Arbor, MI, USA).

HCC2998 cells were treated for 48 h with the vehicle (0.1% DMSO or 0.1% ethanol according to the drugs solubility, see S2 Table) or the indicated drugs. The culture medium, including vehicle and investigated drugs was exchanged every 24 h. After cell harvesting, the CYP2W1 mRNA levels were analyzed as described above. All experiments were performed at least twice in at least two replicates per experiment.

### Statistics

Cyp2w1 mRNA expression was correlated to the methylation percentage at each CpG site and to the average methylation for each amplicon using Spearman correlation coefficient (R_spearman_) for p<0.01. The methylation percentage differences from embryonic (E, E13-E18), early postnatal (PN, D0-D7) to adult ages (D28-13M) were assessed by Kruskal-Wallis in mice and by Mann-Whitney tests between fetal and adult human samples. Two-tailed Student t-test was performed to analyze CYP2W1 fold change in HCC2998 cells. All data were expressed as mean ± SEM and statistical significance was defined as p<0.05 (GraphPad Prism v.5.0; GraphPad Software, La Jolla, CA, USA).

## Results

### CYP2W1 developmental expression

**Fig 1:**
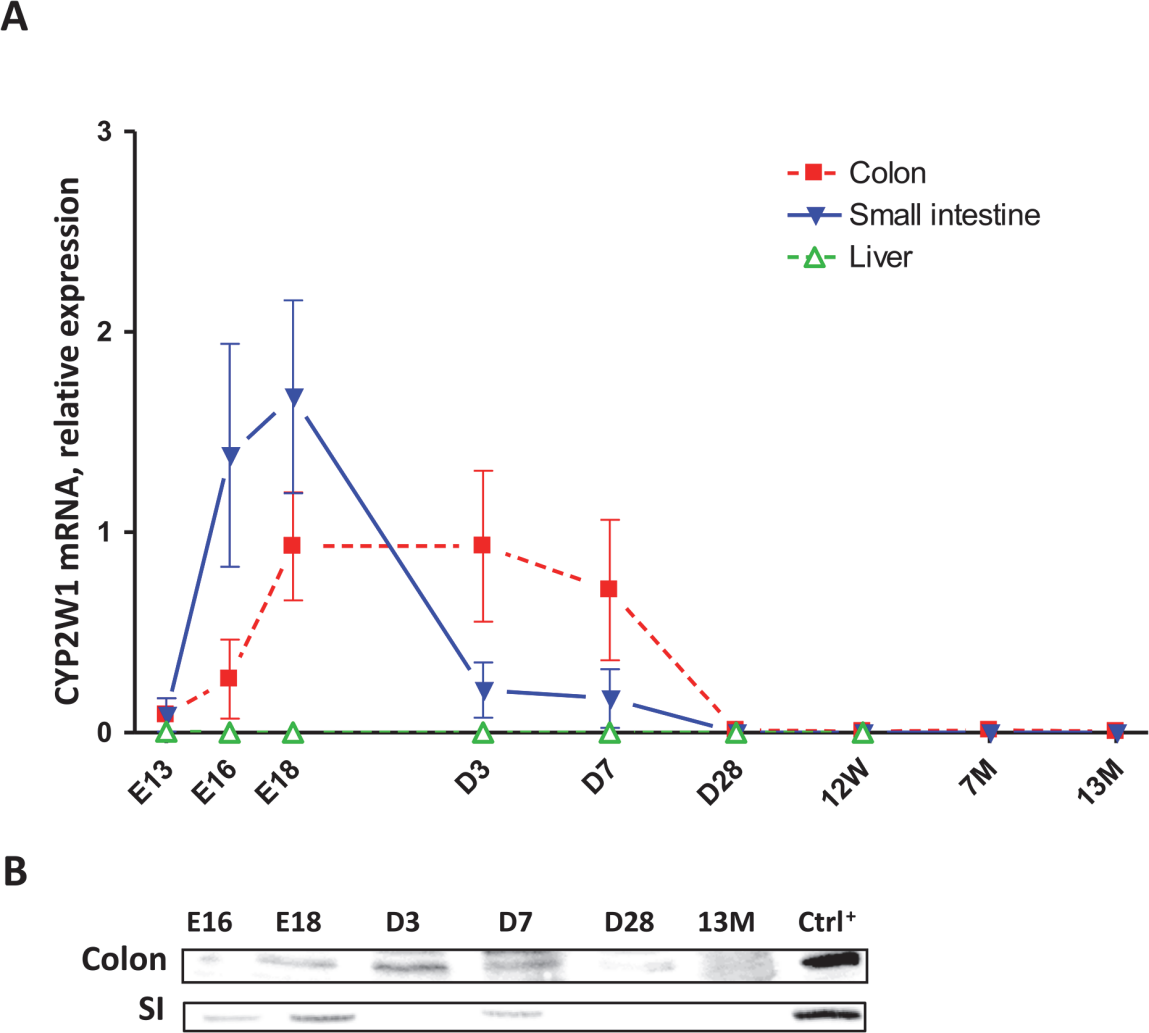
Developmental expression of murine CYP2W1 in the colon and small intestine. A. Mice Cyp2w1 expression change from embryonic day E13 to 13 months of age (n = 6 per time point). Cyp2w1 mRNA levels were normalized against housekeeping genes Tjp1, 18s, Gapdh and arbitrary related to E18 time point. Data are presented as mean±SEM. B. Cyp2w1 protein expression. Representative CYP2W1 immunoblot with equal amount of total protein applied per lane. E, embryonic day, D, day, W, week, M, month of age. Ctrl^+^, murine Cyp2w1 overexpressed in HEK293 cells.

We have studied the CYP2W1 expression on mRNA and protein levels in pre- and postnatal periods in mice and human colon and SI. Cyp2w1 mRNA appears in the mouse embryonic colon and SI at the gestational day 13 (E13). The expression peaks at E18 and is silenced rapidly after the birth (Fig 1A). The expression curves for both tissues are similar, however, they do not match exactly: mRNA expression starts earlier in SI and sharply decreases already by post-natal day three, whereas corresponding levels in colon tissues were stable until post-natal day 7. At four weeks of age (D28), the tissue-specific Cyp2w1 mRNA expression was silenced in both SI and colon tissues. As expected, no Cyp2w1 transcripts were detected in the liver at any time point (Fig 1A). The Cyp2w1 protein data as shown on Fig 1B confirm the mRNA expression pattern in colon and SI.

**Fig 2:**
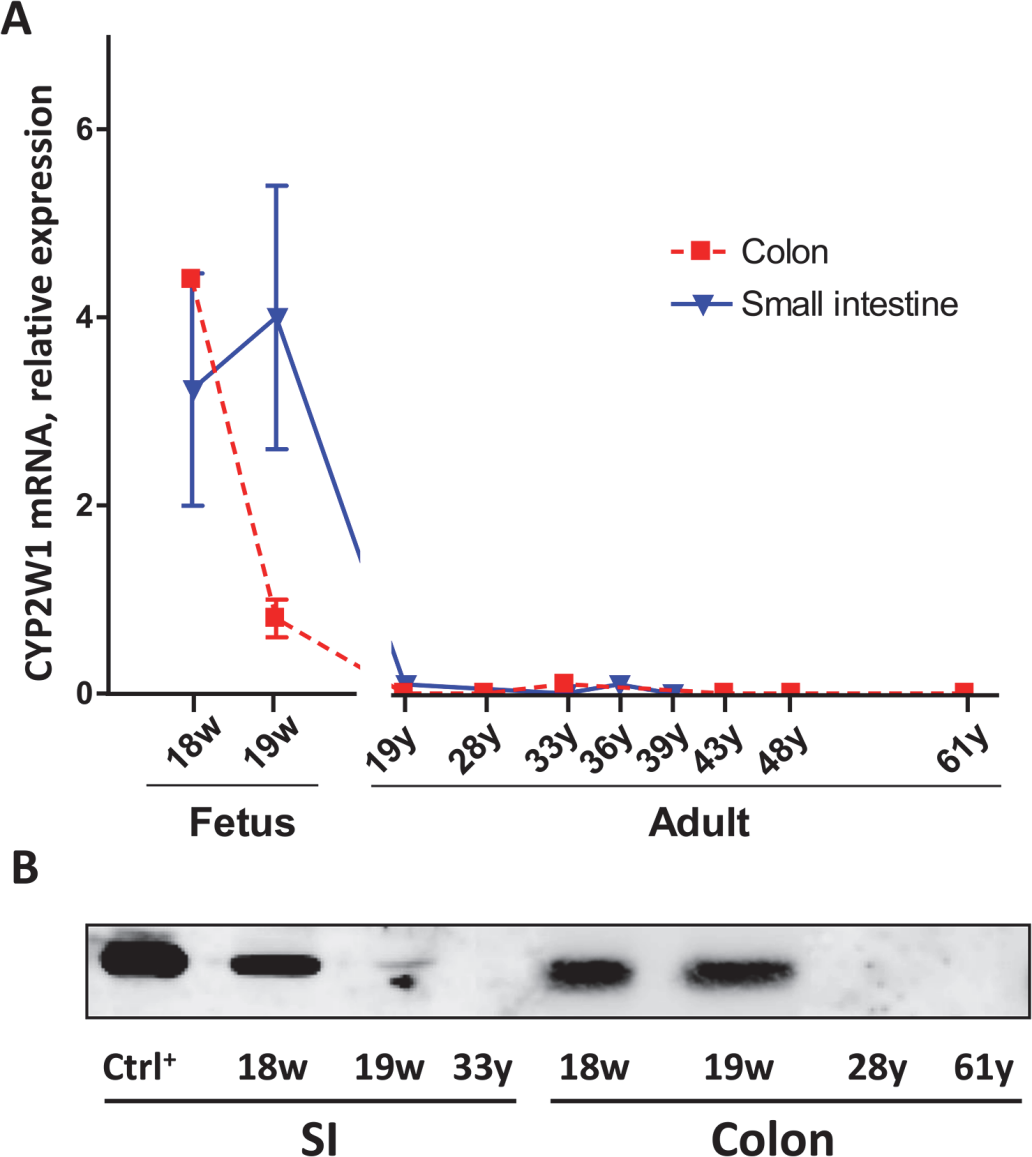
Developmental expression of human CYP2W1 in the colon and small intestine. A. CYP2W1 mRNA levels were normalized against housekeeping genes TBP, EIF2B2 and arbitrary related to the fetal colon value at the gestational week 19. Data are presented as mean±SEM. B. CYP2W1 protein expression. Representative CYP2W1 immunoblot with equal amount of total protein applied per lane. W, gestational week, Y, year of age. Ctrl^+^, CYP2W1 overexpressed in HEK293 cells.

The CYP2W1 mRNA was detected in human fetal colon and SI at the gestational weeks 18–19 but not in any of the adult samples analyzed (Fig 2A). Expression in SI was stable over both studied fetal ages while in colon it started to decline from week 19. Consistent with the murine samples the human protein expression followed the CYP2W1 transcript’s pattern (Fig 2B).

### Immunohistochemistry analysis

**Fig 3:**
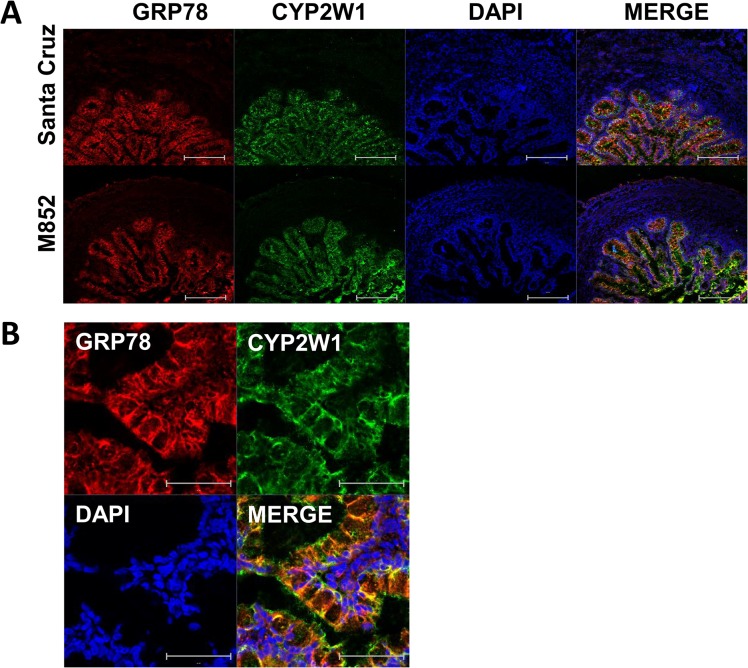
CYP2W1 expression in human fetal colon at gestational week 19. A. Immunohistochemical localization of CYP2W1 in the crypts of the colon section. No CYP2W1 signals were observed in the surrounding tissues. Scale bar = 200 μm. B. Intracellular localization of CYP2W1. The yellow color in the Merge panel indicates co-localization of GRP78 and CYP2W1. Scale bar, 50 μm. Color code: endoplasmic reticulum marker GRP78, red, CYP2W1, green, DAPI, blue.

Immunohistochemical analysis using antibodies from two different sources was used to identify the cells types expressing CYP2W1 in the human fetal colon samples from gestational week 19. CYP2W1 expression was observed in the crypt cells (Fig 3A and 3B) with both antibodies confirming this pattern (Fig 3A). No signals were observed in the cells constituting lamina propria, muscularis mucosae or submucosa (Fig 3A). A typical endoplasmic reticulum expression of CYP2W1 in the crypt cells was confirmed by its co-localization with the ER marker, an abundant molecular chaperone, GRP78 (Fig 3B).

### Epigenetic regulation of CYP2W1

It has been suggested that the expression of CYP2W1 in colon cancer cells requires the demethylation of CpG island in the exon1/intron 1 junction [9]. We hypothesized that a similar mechanism may govern CYP2W1 gene expression during the development.

**Fig 4:**
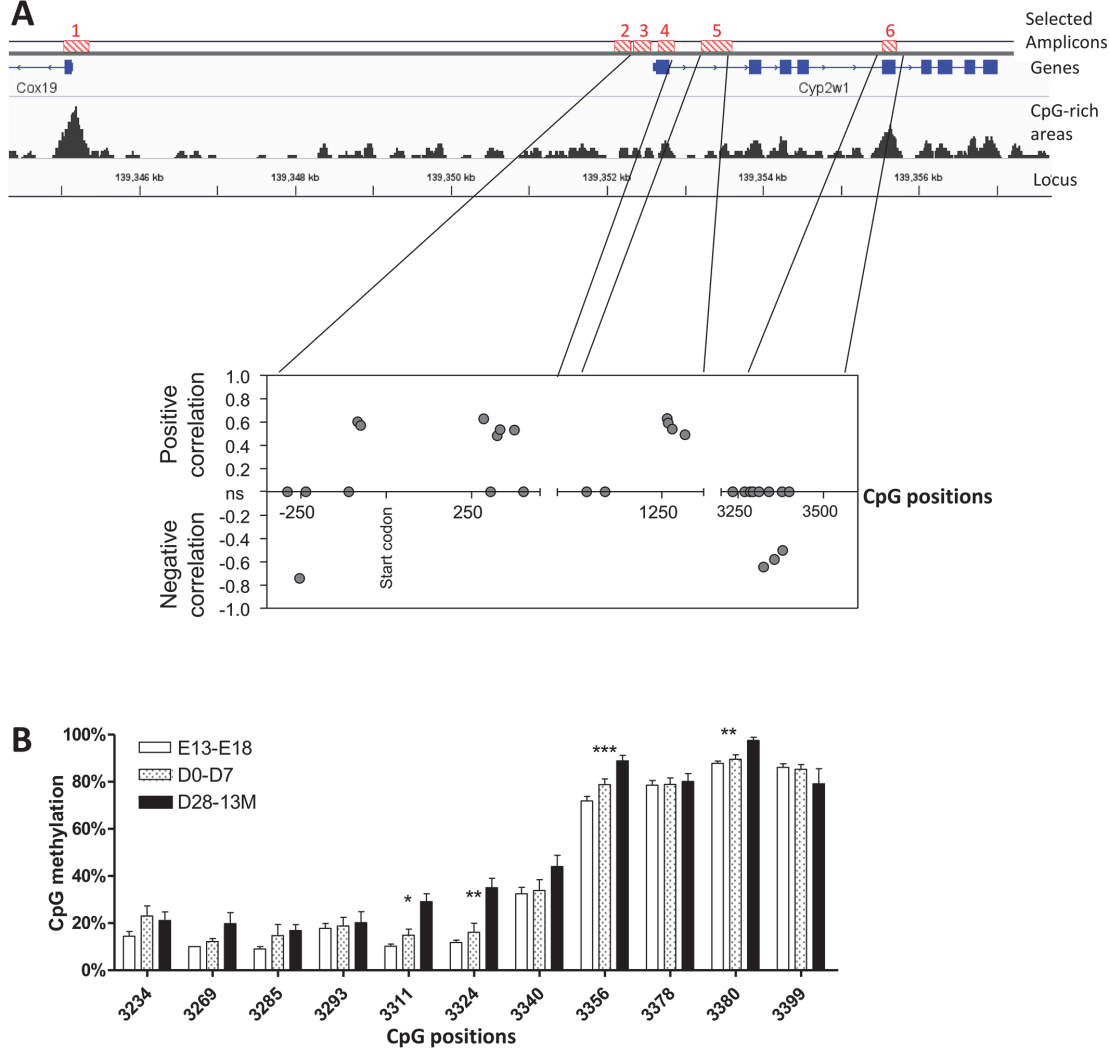
Methylation state of murine CYP2W1 in pre- and postnatal colon. A. Top panel. The figure shows 6 selected amplicon sites (red striped boxes, top), the genes including exons (blue boxes) and introns (blue lines) as well as the CpG-rich areas (grey peaks, bottom) within approximately 13 kb upstream of the murine Cyp2w1 transcriptional start site. Bottom panel. Murine Cyp2w1 mRNA expression change in colon from embryonic day E13 to 13 months post-natal were correlated to the percentage of CpG methylation of the 6 amplicons. One CpG site in the promoter shows a negative correlation with the Cyp2w1 gene expression whereas most of the CpG sites within the gene show a positive correlation. Exon 5, in contrary, displays a strong negative correlation. The X axis shows the CpG localization along the gene and the Y axis shows the R_spearman_ for p<0,01. Of note, four additional CpG sites had p = [0,5–0,1] and, therefore, were considered as non-significant (ns). B. Methylation dynamics in exon 5. Kruskal-Wallis tests within the age groups by CpG site were performed. E, embryonic day, post-natal: D, day, W, week, M, month of age. * p<0.05, ** p<0.01, ***p<0.001. Data are presented as mean±SEM.

Scanning of the regulatory and gene coding regions of murine *Cyp2w1* gene could not locate CpG islands that are characteristic for the human gene. However, recent analyses attached an important role also for CpG sites outside the canonical CpG islands [21]. Six of such areas (amplicons) with the relatively high CpG density from -13 kb to the *Cyp2w1* gene were selected for further analyses (Fig 4A, upper panel). The percentage of methylated cytosines in these amplicons was evaluated in the genomic DNA isolated from mouse colon tissue. Fig 4A (bottom panel) presents significant Spearman correlation (p<0.01) between CpG methylation percentage and Cyp2w1 mRNA expression. Four additional CpG sites had R_spearman_ p = [0,5–0,1] and, therefore the correlations were considered as non-significant. Such correlation was not found in the other amplicons and the distal promoter was consistently demethylated in all samples (data not shown).

In addition, the statistically significant differences in murine Cyp2w1 methylation levels between the different age groups (from adult to fetal age) were observed at a number of CpG sites. The major differences were seen in the close promoter, exon 1/intron 1, intron 1 and exon 5. Hypomethylation of CpGs in fetal samples was observed at one site in the promoter (-254 bp) and in exon 5 (3311, 3324, 3356, 3380 bp, amplicon 6) (Fig 4B).

**Fig 5:**
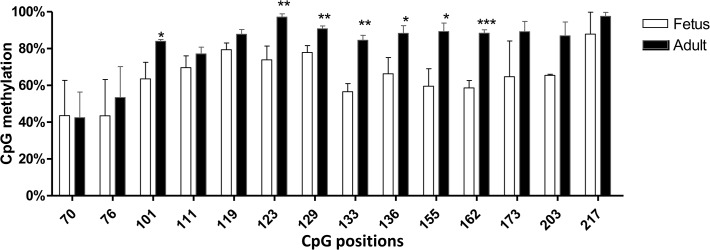
Methylation state of human CYP2W1 in pre- and postnatal colon. Methylation dynamics in exon 1/intron 1. Mann-Whitney tests within the age groups by CpG site were performed. * p<0.05, ** p<0.01, ***p<0.001. Data are presented as mean±SEM.

The methylation state of the human CYP2W1 gene in pre- and post-natal human colon samples was analyzed using the same methodology. We chose the exon 1/intron 1 region, demethylation of which was previously shown to correlate with the expression of CYP2W1 in colon tumors. The methylation of six different CpG sites in this region was found to be significantly higher in adult samples as compared to the fetal colon (Fig 5). This is consistent with the methylation state of the gene as induced by CRC [9].

### Drug-induced regulation of CYP2W1 expression

**Fig 6:**
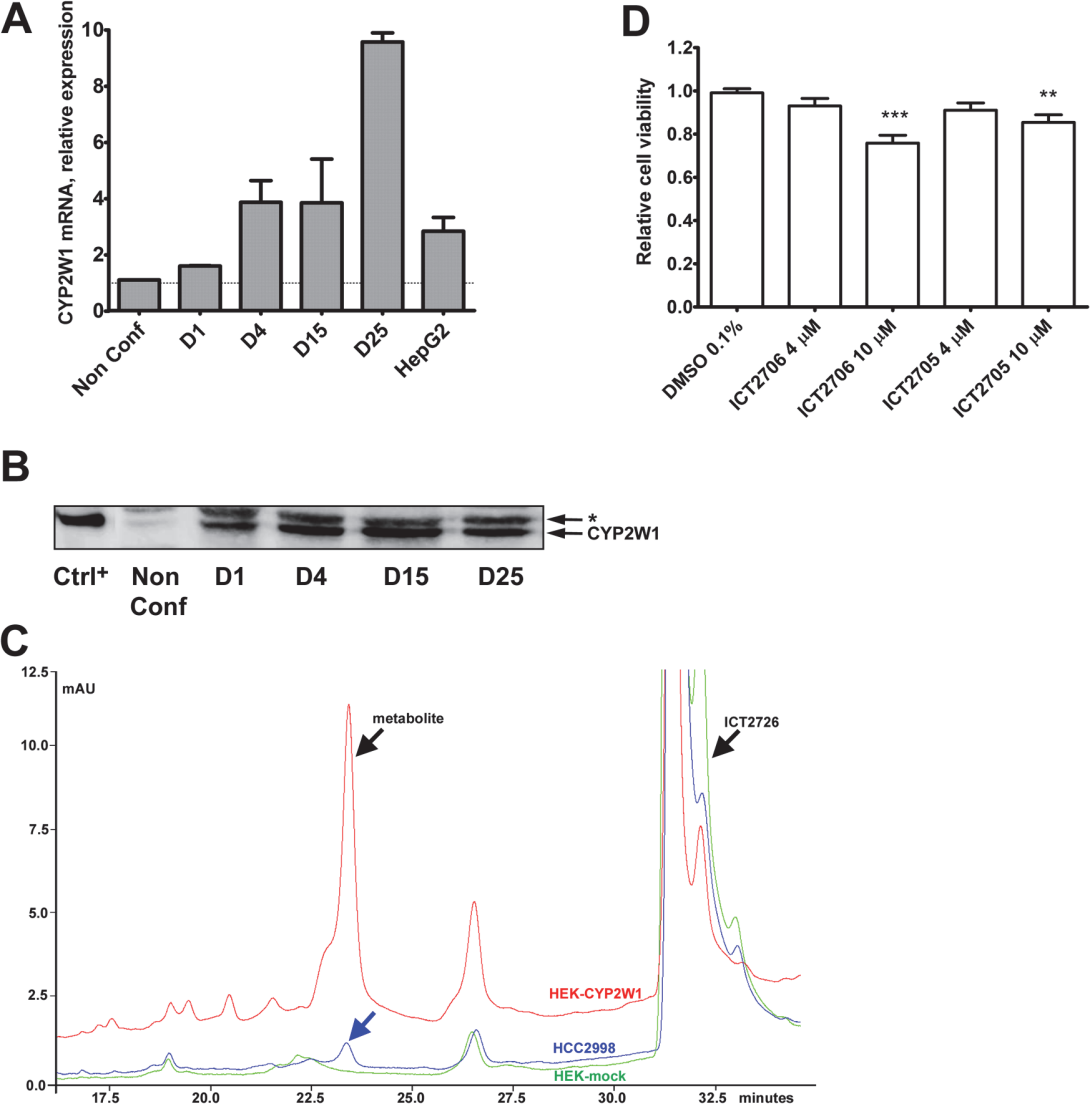
Endogenous expression of CYP2W1 in the colon adenocarcinoma cell line HCC2998. A. CYP2W1 mRNA expression. The CYP2W1 mRNA was normalized by housekeeping genes PPIA, EIF2B2 and non-confluent cells were arbitrary taken as a reference. Data are presented as mean±SEM. B. CYP2W1protein expression. Representative immunoblot with equal amounts of total protein applied per lane. NonConf, non-confluent cells, D, days of culturing after cells reached the confluent state. All experiments were performed at least twice in at least two replicates per experiment. Ctrl^+^, CYP2W1 overexpressed in transfected HEK293T cells, *, non-specific band. C. Catalytic activity of CYP2W1. A representative chromatogram of HCC2998 (blue), CYP2W1 overexpressed in transfected HEK293T (red) or mock cells (green) activity, respectively. Cells were incubated for 24 h with the CYP2W1-specific substrate ICT2726. The metabolite peak is observed at 23 min and the substrate at 32 min. All experiments were performed at least twice in at least two replicates per experiment. D. Cytotoxic effect of duocarmycin prodrugs on HCC2998 cells. Confluent colon adenocarcinoma cells HCC2998 were incubated for 72 h with ICT2706 and ICT2705. Cell viability was assessed by EZ4U assay. Data are presented in relation to the cell viability of vehicle treated cells as mean±SEM of three experiments. ** p<0.01, ***p<0.001.

Increased expression of CYP2W1 can be beneficial for the specific prodrug based treatment of cancer (see Introduction). However, an appropriate colon cancer cell model with a constitutive CYP2W1 expression suitable for the *in vitro* regulation studies has hitherto not been identified. Scanning of a number of colon adenocarcinoma cell lines (results not shown) led to identification of the HCC2998 cell line (National Cancer Institute, Frederick, USA) with a significant level of CYP2W1 expression and activity. The HCC2998 cells express similar or even higher amounts of CYP2W1 than HepG2 cells (Fig 6A), a hepatoma cell line with constitutive expression of CYP2W1 [4]. Interestingly, the CYP2W1 mRNA and protein levels in these cells were substantially increasing upon prolonged cultivation for 4–25 days after the cells reached the confluent state (Fig 6A and 6B). Therefore, all subsequent analyses were done on confluent cells, which were then treated with drugs for additional five days.

CYP2W1 expressed in HCC2998 cells appears to be catalytically active as it was found to metabolize the CYP2W1 specific substrate, non-toxic duocarmycin derivative ICT2726 [11]. Analyses of these cells revealed formation of ICT2726 metabolite with the same retention time as in the positive control, HEK 293T cells overexpressing CYP2W1 (Fig 6C).

Moreover, incubation of HCC2998 cells with the duocarmycin prodrugs ICT2706 and ICT2705 that were shown to be converted by CYP2W1 into potent cytotoxins [11] led to a significant decrease of cell viability (Fig 6D).

**Fig 7:**
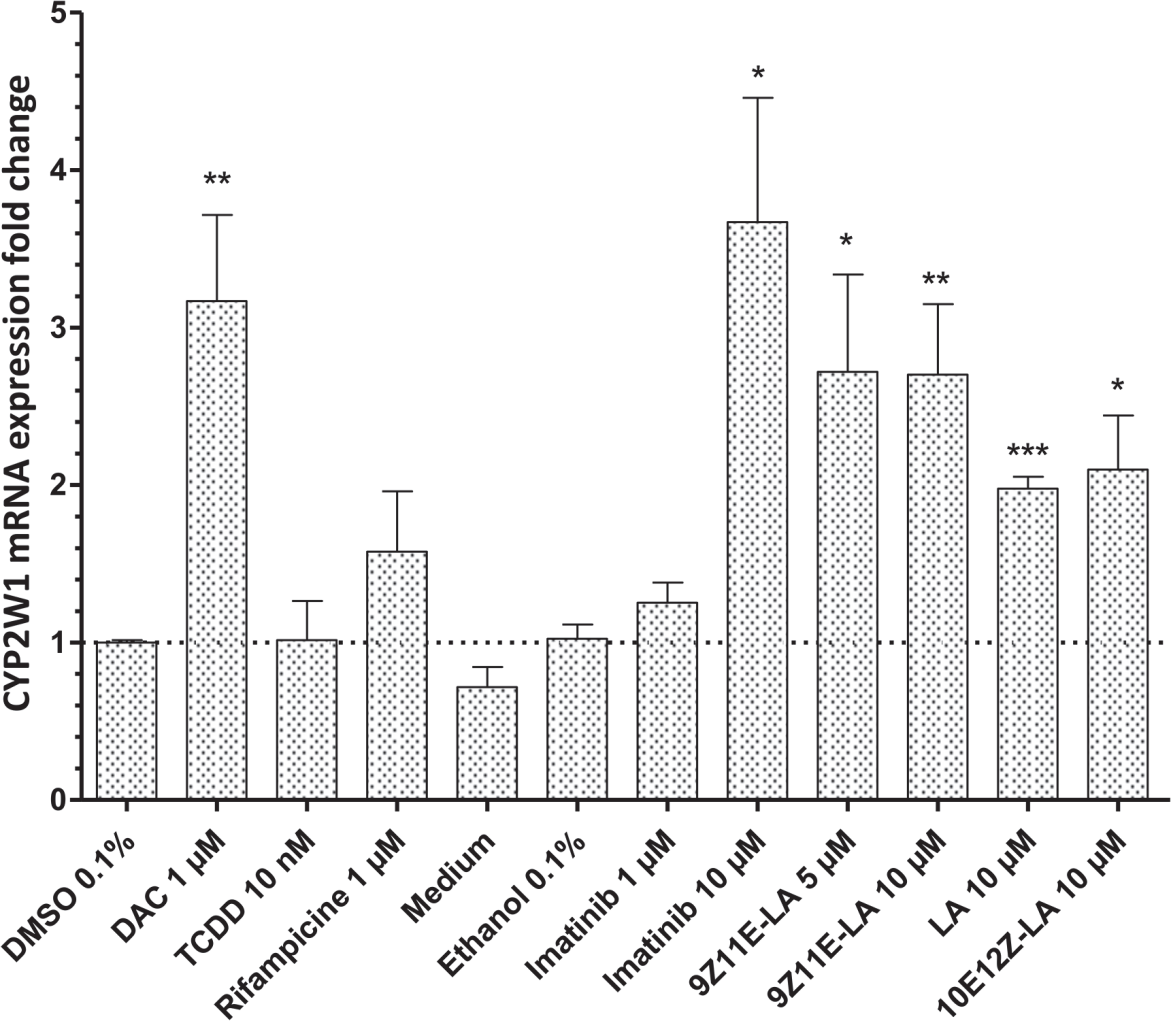
Screening of CYP2W1 inducers in the colon adenocarcinoma cell line HCC2998. CYP2W1 mRNA levels following the 48 h treatment with several selected drugs. The CYP2W1 transcript expression was normalized by housekeeping genes EIF2B2, HMBS and PPIA and related to control (vehicle alone, either DMSO or ethanol depending on the drug). Data are presented as mean±SEM of at least two experiments in at least two replicates per experiment. * p<0.05, ** p<0.01, ***p<0.001.

Using the HCC2998 cell model, we examined a series of biologically active substances for the induction of CYP2W1 expression. Treatment of the cells with DAC, imatinib, LA and the two conjugated (9Z11E-LA, 11E12Z-LA) significantly increased the CYP2W1 mRNA levels (p<0.05) as compared to vehicle treated cells (Fig 7). In order to test whether this effect might be determined by the drug mediated demethylation of CYP2W1 we tested the methylation state of the same CpG sites in the exon 1/intron 1 region of the human *CYP2W1* gene (CpG positions 70, 76, 101, 111, 119, 123, 129, 133, 136, 155, 162, 173, 203, 217) in the cells treated with indicated drugs or vehicle. The percentage of methylated cytosines in all of these CpG sites was found unaltered between the drug treated and control cells (data not shown).

Induction of CYP1A1 was also observed by the previously reported CYP1A1 inducers, citco, TCDD and DAC [22–24] (S2 Fig) validating thus our cell induction model. No induction of CYP2W1 or CYP1A1 was observed by other drugs used in this study (see Methods) including previously reported CYP1A1 inducer GW-610 [14].

## Discussion

CYP2W1 is one of the enigmatic P450 isoforms characterized by the barely detectable expression in adult organisms and unknown endogenous function in development. At the same time cancer-specific expression and discovery of prodrugs metabolized by CYP2W1 to cytotoxins makes it an interesting target for cancer therapy and therefore a subject for intensive studies on its regulation and function in ontogeny and neoplasia.

Here we provide with information on the developmental pattern of CYP2W1 expression in mice and humans and the epigenetic regulation, which characterizes CYP2W1 as a novel oncofetal gene. In addition, screening of a broad spectrum of biologically active molecules yielded a list of potential CYP2W1 inducers that might be instrumental for its up-regulation in cancer tissues, increasing thus the efficacy of prodrug therapy.

Embryonic expression of Cyp2w1 in rodents has been suggested previously, albeit only at mRNA level. The data were somewhat inconclusive due to a large standard deviations reported in the rat study [4] and the use of pooled samples of different murine tissues [25]. Here, we show in detail the expression of Cyp2w1 mRNA and protein in fetal murine colon and in SI. It should be noted that the developmental expression curves in these tissues do not overlap: Cyp2w1 mRNA is detected earlier in SI than in colon. This might indicate synchronization with the development of the whole GI tract. However the colon expression remains at a plateau for a longer period of time. In both tissues, gene silencing occurred within the first 28 days of post-natal life.

Information on the human embryonic expression of CYP2W1 is scarce. Using a human fetal mRNA MTC panel lacking GI tissues, Choudhary *et al*. found qualitatively a transient CYP2W1 mRNA expression in lungs, liver, skeletal muscle and kidney at gestational week 30 [25]. Here, we could describe the CYP2W1 mRNA and protein levels of CYP2W1 in human fetal colon and in SI, and using the immunohistochemical analysis, show the regio-selective distribution of CYP2W1 in the crypt cells of embryonic human colon, reminiscent of the CYP2W1 expression in colon cancer cells [7].

Tumorigenesis and embryogenesis are suggested to share common pathways [12,13]. A number of CRC biomarkers and cancer therapy target genes, such as carcinoembryonic antigen (CEA) [26], Coding Region Determinant-Binding Protein [27] and Cripto-1 [28], Trophoblast glycoprotein (5T4) [29] are shown to be regulated in such manner, i.e. expressed in tumors, whereas their expression in normal tissues is mainly limited to pre-natal period. Moreover, an overlap between murine gut developmentally regulated genes and those reactivated in human CRC tumors was reported previously, including for instance the insulin-like growth factor II (IGF-II), which is known to enhance tumor growth and suppress apoptosis [30].

Among the P450s, similar developmentally regulated expression pattern was reported for CYP1B1, which is involved in eye development [31] and is also expressed in various primary tumors and metastases, while its expression is generally undetectable in corresponding normal cells [32,33]. Likewise, CYP1A1 gene is highly expressed in several tumors but much less in corresponding normal cells [34].

One important mechanism controlling the expression of oncofetal genes is epigenetic regulation, the CpG methylation in particular. Here we present detailed data on the methylation state of murine and human *CYP2W1* in GI tissues from fetus to adult age. The developmental shift from hypomethylation to hypermethylation in the exon 1/intron 1 CpG island of human *CYP2W1* correlates with the gene expression levels of CYP2W1 and is perfectly consistent with similar changes found in normal versus colon tumor tissues [9]. Interestingly, such methylation shifts in the promoters of many intestine-specific genes, including Cyp2w1 were recently related to intestine maturation in preterm pig [35].

The methylation state of genomic regions outside of CpG islands and the canonical promoter sites as shown in the mouse *Cyp2w1* gene (Fig 4A) is receiving increased attention [21]. Recent findings revealed positive correlation between intragenic DNA methylation level and gene expression [21,36–38].

In the whole-genome DNA methylation and gene expression study of leukemia Kulis *et al*. showed significant both positive and negative correlation of methylation status and gene expression in the gene body (intragenic) of approximately 900 genes with absence of methylation changes in the promoters [21]. Similar studies on cell cultures demonstrated that the lowest level of intragenic methylation was correlated with both the lowest and highest gene expression [39]. Such intragenic methylation is suggested to regulate gene expression by promoting alternative promoters, enhancer activation, transcript elongation or regulating intragenic non-coding RNA [21].

Transcriptional regulation of gene expression is commonly studied in the cultured cell lines with the constitutive expression of the gene of interest. The colon adenocarcinoma cell line HCC2998 used in this study seems to be an adequate model in that respect as it constitutively produces relatively high amounts of functional CYP2W1, the CYP2W1 levels are also increasing with the enhanced cell density. Similar density-dependent upregulation of CYP3A4 was observed in post-confluent human hepatoma cells [40,41] and in adenocarcinoma cell line Caco2-TC7 [42], apparently due to cell adhesion triggered re-differentiation of these cells.

The HCC2998 cell model was successfully used to test whether the CYP2W1 expression can be exogenously induced, which might be useful for augmenting the effects of CYP2W1-dependent cancer therapy [11]. Among the tested substances, imatinib, DAC and LA were found to be the most potent inducers of CYP2W1 expression. The highest induction was found in cells treated with imatinib, which was previously known as a P450 inhibitor [43]. However, an unpublished transcriptomic analysis of leukemia cells after imatinib treatment (Geo Dataset: GDS3044) also revealed induction of CYP2W1 in line with our data. DAC-mediated CYP2W1 induction has previously been reported in Caco2-TC7 cells [4]. DAC is a commonly used demethylating agent and therefore similar mechanism of CYP2W1 induction cannot be ruled out.

CYP2W1-mediated metabolism of certain lipids including arachidonic acid and lysolecithin and their stereoisomers was reported earlier [4,20]. In our study CYP2W1 was significantly induced by LA and the 2 conjugated-LA, however, no stereoselectivity was found.

Is has previously been reported that the *in vitro* anti-cancer effects of 9Z11E-LA and 10E12Z-LA isomers [44,45] involve numerous pathways, including PPARγ [46], CAR [47] or ERα pathway [48]. In our model, the CAR and PPARγ agonists citco and ciglitazone, respectively did not show any induction. Therefore, in HCC2998 cells, the CYP2W1 induction may involve other, yet unknown mechanisms. These, most probably do not include epigenetic regulation, the mechanism that governs the developmental expression of CYP2W1, as neither of the CYP2W1 inducing drugs had any effect on the methylation state of CpG sites in the critical exon 1/intron 1 region of the *CYP2W1* gene. It is likely that the CYP2W1 expression is mediated by the nuclear proteins that are activated in a phosphorylation cascade alteration induced by the treatment of the tyrosine kinase inhibitor imatinib. The details of this signal transduction mechanisms remains to be elucidated. It can be also speculated that the endogenous function of CYP2W1 might be connected with the lipid metabolism.

It has been previously suggested that AhR may mediate the CYP2W1 induction [4]. However, despite the strong induction of CYP1A1 by TCDD, a potent AhR ligand and CYP1A1 inducer [49], CYP2W1 expression remained unaffected (Figs 7 and S2). Interestingly, in addition to TCDD, the CYP1A1 expression was upregulated also by imatinib, which is to our knowledge the first report of such induction (S2 Fig). Taken together with the CYP1A1 involvement in the metabolism of imatinib [50] and in activation of other antitumor agents (Phortress), this finding might be of a clinical relevance and should be taken in account upon combined treatment with imatinib and other CYP1A1 metabolized drugs.

In conclusion we show the developmental expression of CYP2W1 in the GI tract, to a great extent mediated by epigenetic modifications, its specific location, and regulation by anticancer drugs which can be considered as an adjuvant therapy of colon cancer metastases and hepatic cancers in the future. Additional information is required regarding the mechanisms of specific regulation of the enzyme in primary colon cancer tumors.

## Supporting Information

S1 Fig CpG rich areas of the murine cyp2w1 gene. The murine cyp2w1 gene, its upstream region and CpG positions. The figure shows 6 selected amplicon sites (red boxes, top), the genes including their exons (blue boxes) and introns (blue lines) as well as the CpG-rich areas (grey peaks, bottom) within ∼ −13 kb upstream of the Cyp2w1 transcriptional start site.
(TIF)

S2 Fig Screening of CYP1A1 inducers. CYP1A1 mRNA levels following the 48 h treatment of HCC2998 cells with selected drugs. The CYP1A1 transcript expression was normalized by EIF2B2, HMBS and PPIA and compared to control cells (vehicle alone, either DMSO or ethanol depending of the solubility). All experiments were performed at least twice in at least two replicates per experiment. Mean and SEM are shown. * p<0.05, **p<0.01
(TIF)

S1 Table PCR conditions for epigenetic study. PCR conditions and primer sequences. 50 ng bisulfite converted DNA was amplified by 32 cycles of 94°C for 30 sec, 48°C-60°C annealing temperature (indicated as Temp) for 30 sec, and 72°C for 1min. Taq = Taq DNA polymerase (Thermo Scientific, Germany), Kapa = KAPA HiFi HotStartUracil ReadyMix (Kapa Biosystems, USA). MgCl_2_ = volume of 25 mM MgCl_2_ in a 30 μl reaction.
(TIF)

S2 Table Potential inducers of CYP2W1 and CYP1A1. (TIF)
