# Supplementary figures and images for: Developmental Regulation and Induction of Cytochrome P450 2W1, an Enzyme Expressed in Colon Tumors
Source: PLoS One. 2015 Apr 6;10(4):e0122820. doi: 10.1371/journal.pone.0122820 (PMC4386763; doi:10.1371/journal.pone.0122820)

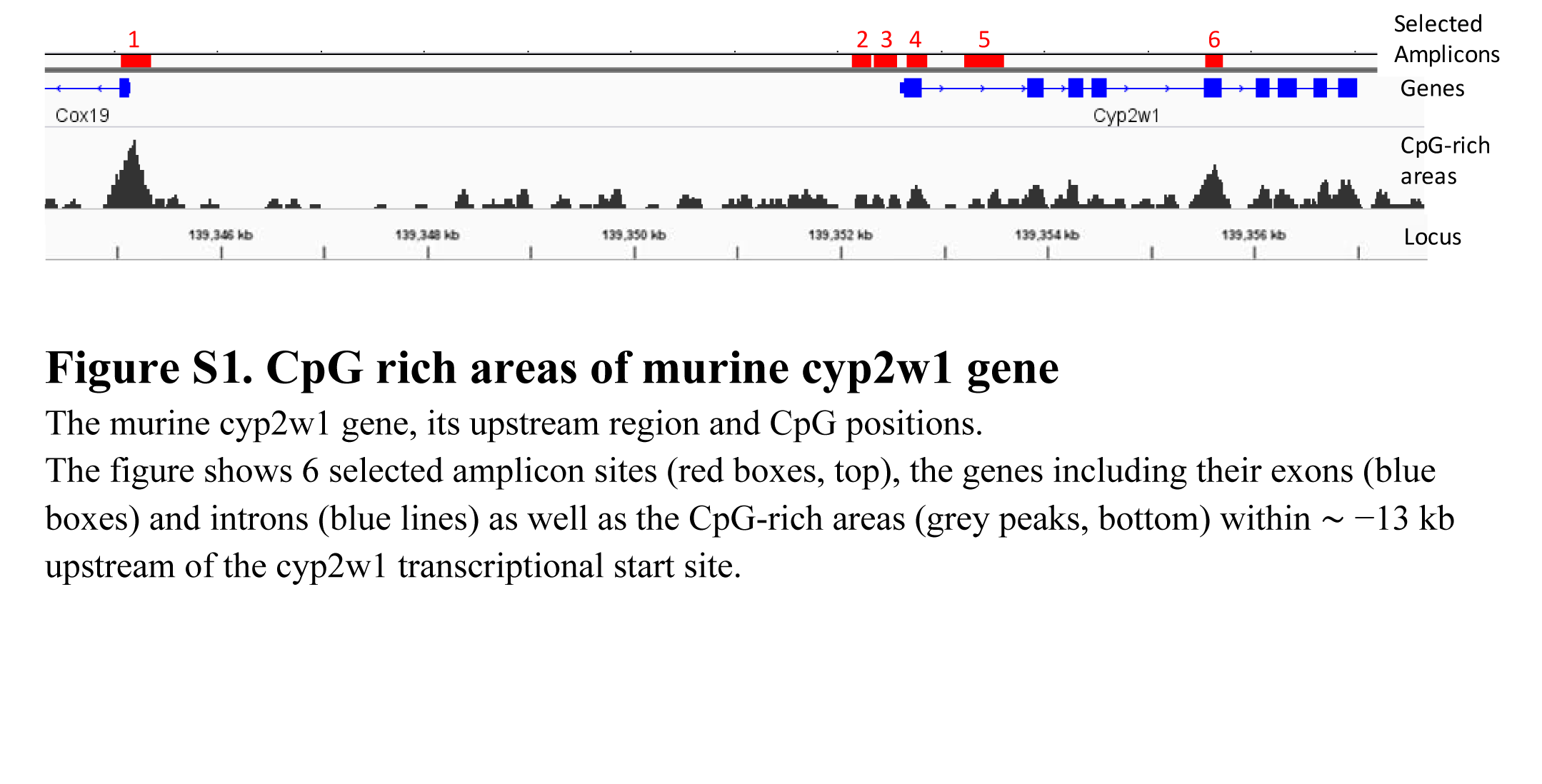

Supplement: S1 Fig — The murine cyp2w1 gene, its upstream region and CpG positions. The figure shows 6 selected amplicon sites (red boxes, top), the genes including their exons (blue boxes) and introns (blue lines) as well as the CpG-rich areas (grey peaks, bottom) within ∼ −13 kb upstream of the Cyp2w1 transcriptional start site. (TIF) [file pone.0122820.s001.tif]

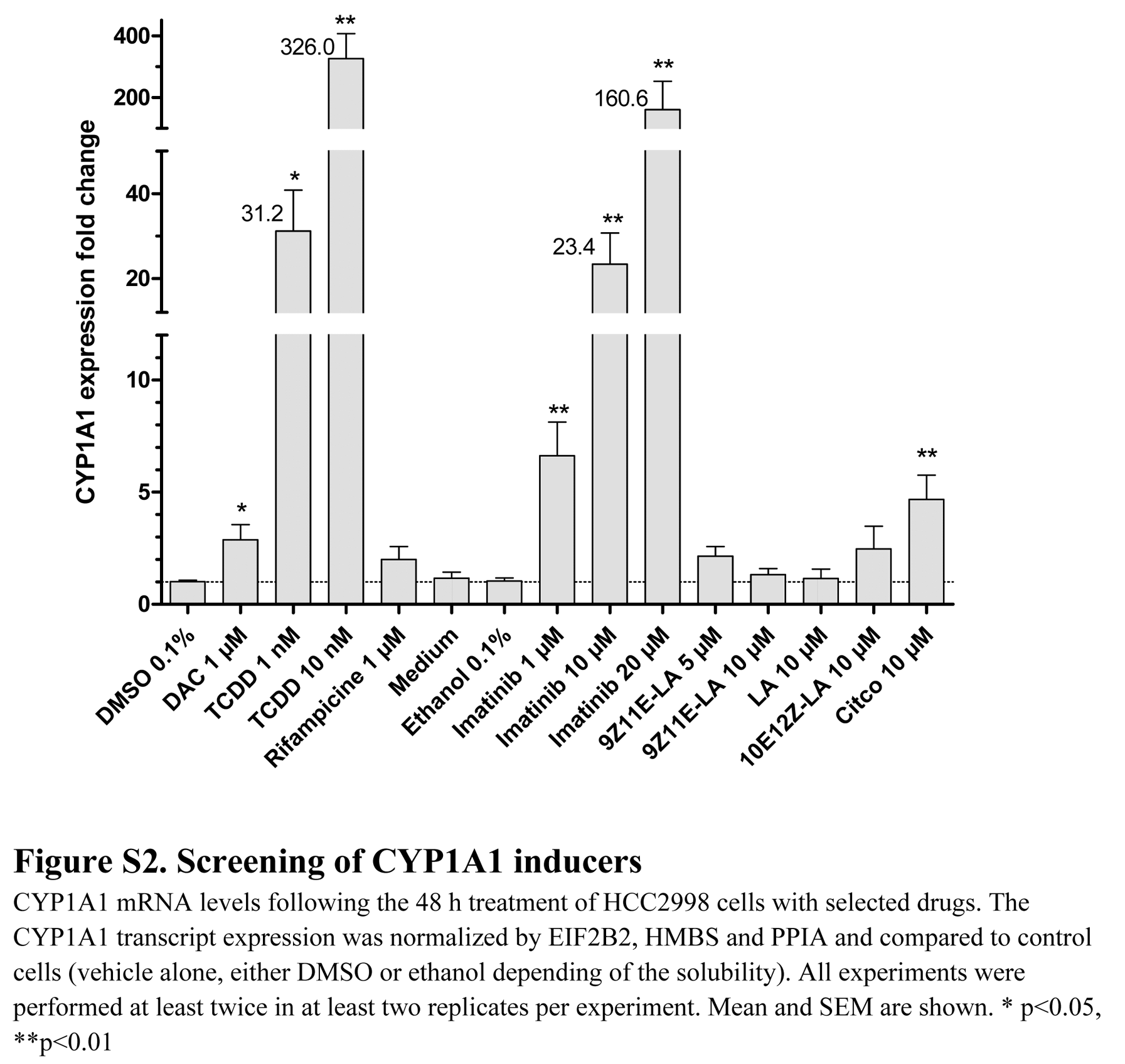

Supplement: S2 Fig — CYP1A1 mRNA levels following the 48 h treatment of HCC2998 cells with selected drugs. The CYP1A1 transcript expression was normalized by EIF2B2, HMBS and PPIA and compared to control cells (vehicle alone, either DMSO or ethanol depending of the solubility). All experiments were performed at least twice in at least two replicates per experiment. Mean and SEM are shown. * p<0.05, **p<0.01 (TIF) [file pone.0122820.s002.tif]

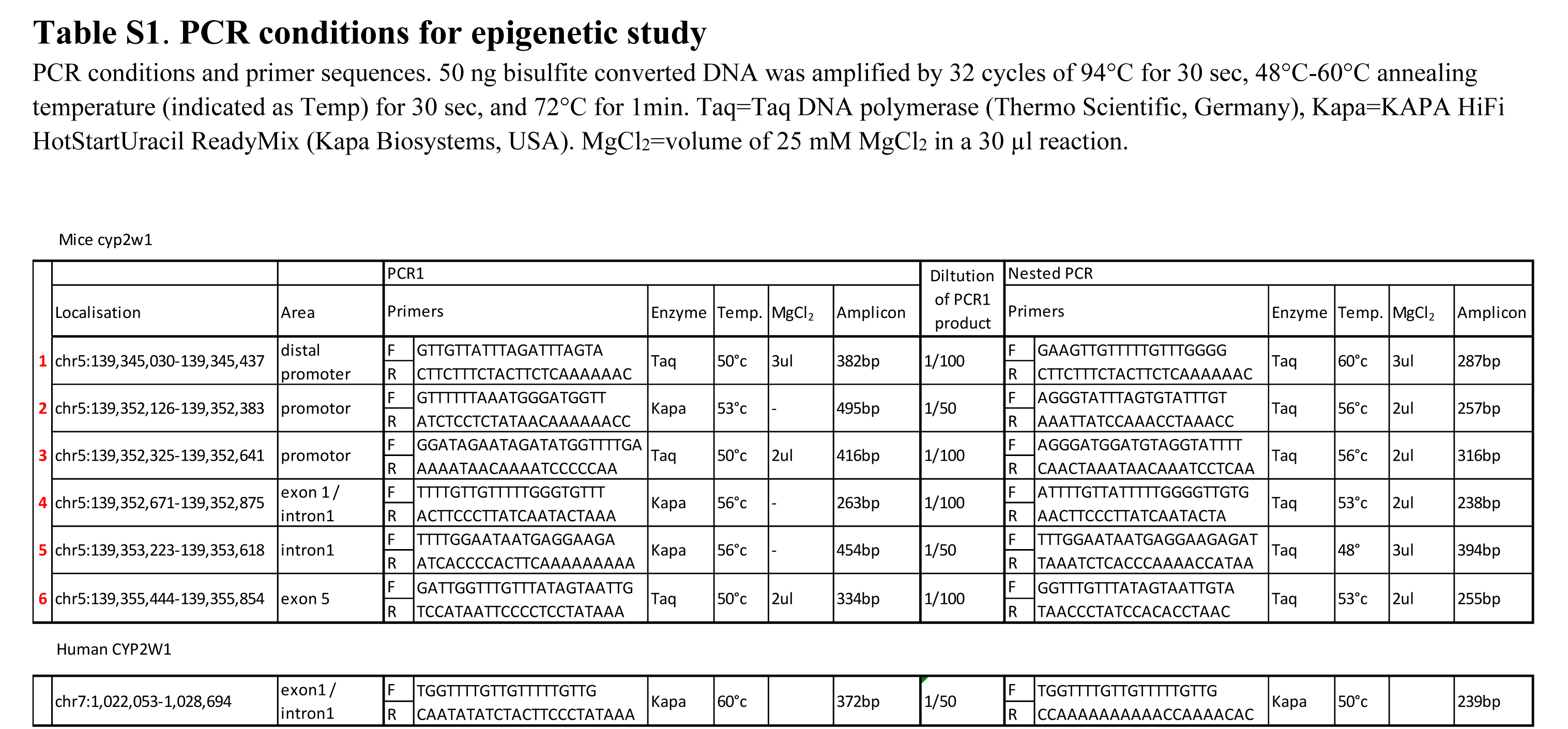

Supplement: S1 Table — PCR conditions and primer sequences. 50 ng bisulfite converted DNA was amplified by 32 cycles of 94°C for 30 sec, 48°C-60°C annealing temperature (indicated as Temp) for 30 sec, and 72°C for 1min. Taq = Taq DNA polymerase (Thermo Scientific, Germany), Kapa = KAPA HiFi HotStartUracil ReadyMix (Kapa Biosystems, USA). MgCl2 = volume of 25 mM MgCl2 in a 30 μl reaction. (TIF) [file pone.0122820.s003.tif]

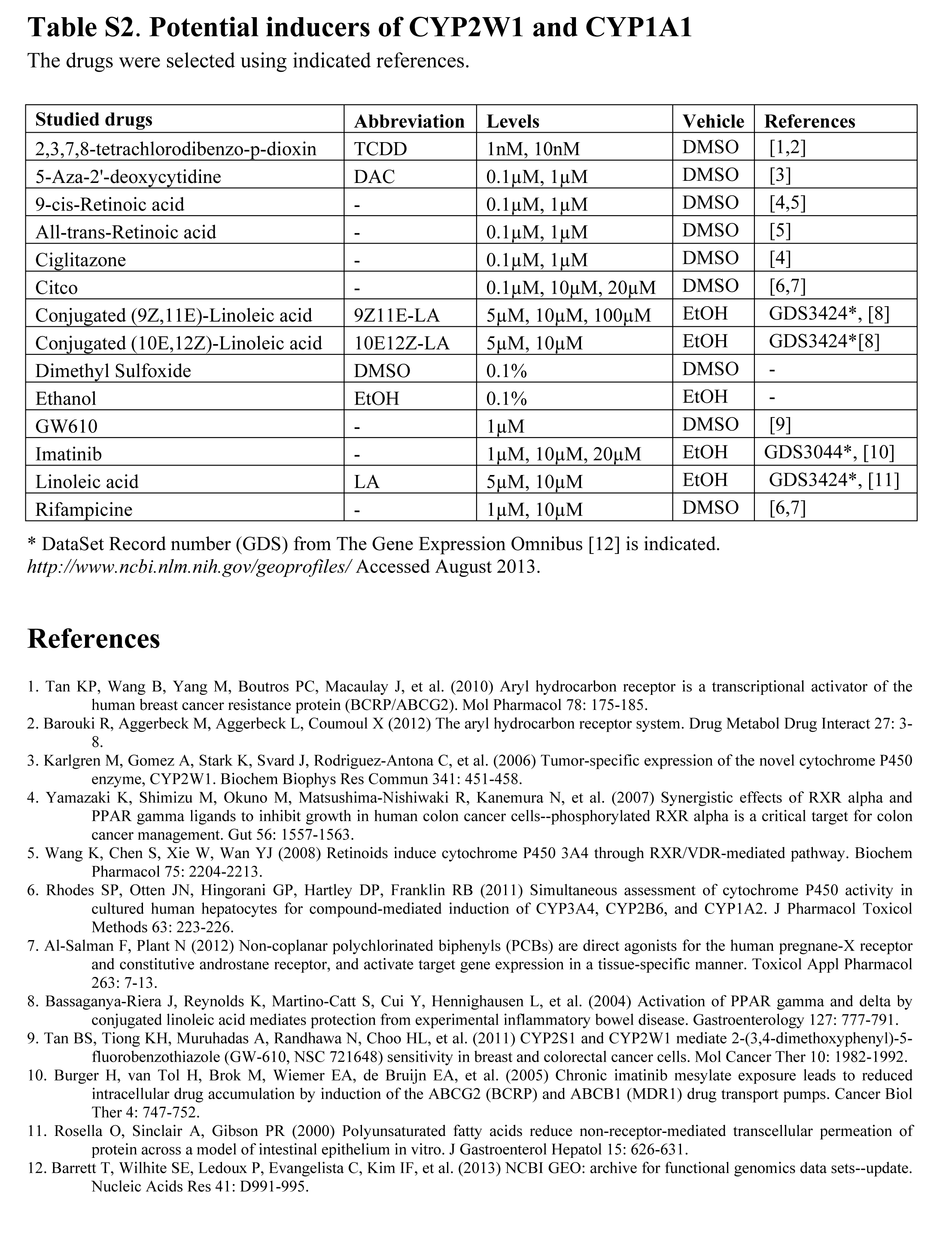

Supplement: S2 Table — (TIF) [file pone.0122820.s004.tif]
